# Single large-scale mitochondrial DNA deletion syndromes: scientific and family conference optimizes the collection of rare disease research outcomes

**DOI:** 10.1186/s13023-025-03632-4

**Published:** 2025-08-04

**Authors:** Laura E. MacMullen, Elizabeth Reynolds, Marissa Weis, Ibrahim George-Sankoh, Sara Nguyen, Katelynn D. Stanley, Mariya Redko, Maria Poblete, Amy C. Goldstein, Rebecca D. Ganetzky

**Affiliations:** 1 Mitochondrial Medicine Frontier Program, Division of Human Genetics, Department of Pediatrics, Children’s Hospital of Philadelphia, Philadelphia, PA 19104 USA; 2 The Champ Foundation, 4711 Hope Valley Road 4F PMB 1171, Durham, NC 27707 USA; 3 Department of Pediatrics, The Perelman School of Medicine, University of Pennsylvania, 3400 Civic Center Blvd, Philadelphia, PA 19104 USA; 4 Center for Computational Medical Genomics, Children’s Hospital of Philadelphia, Philadelphia, PA 19104 USA

**Keywords:** Kearns Sayre syndrome, Pearson syndrome, Chronic progressive external ophthalmoplegia, Single large‐scale mitochondrial deletion syndromes, mtDNA, Rare disease, Conference, Patient-reported outcomes

## Abstract

**Background:**

The SLSMDS Research Network is a collaborative network comprising patient advocates, researchers, clinicians, and affected families seeking to improve outcomes for individuals with single large-scale mitochondrial DNA deletion syndromes (SLSMDS). Building off of jointly developed research infrastructures, including a patient registry and natural history study, advocates and clinicians cohosted the SLSMDS Family and Scientific Conference, enabling the collection of patient data from an ultra-rare and geographically dispersed patient population. Here we describe the data collection procedures for single-time point laboratory assessments and patient reported outcomes for a subset of individuals with SLSMDS.

**Results:**

Utilizing a reproducible model of rare disease data collection, we expand our understanding of the common psychiatric manifestations, describe variability in terms of self-care and quality of life, and emphasize potential biomarkers for individuals with SLSMDS.

**Conclusion:**

Our study describes how efficient patient-researcher partnerships can develop and sustain novel mechanisms to collect rare disease data, improve our understanding of the natural history of these disorders, and support development of future treatments.

**Supplementary Information:**

The online version contains supplementary material available at 10.1186/s13023-025-03632-4.

## Background

Single large-scale mitochondrial DNA deletion syndromes (SLSMDS) are a spectrum of rare, progressive, and multisystemic mitochondrial diseases, including the named entities Pearson syndrome (PS), Kearns Sayre syndrome (KSS), and Chronic Progressive External Ophthalmoplegia (CPEO) [1, 2]. There is currently no effective treatment or cure for SLSMDS.

The SLSMDS research network is a collaborative effort between patient advocates, researchers, clinicians, and affected families, and seeks to improve outcomes for individuals with SLSMDS. The research network is led by The Champ Foundation, a SLSMDS patient advocacy group, in close collaboration with the Children’s Hospital of Philadelphia (CHOP), one of the SLSMDS clinical Centers of Excellence. One of the primary goals of the SLSMDS Research Network is to facilitate patient-centered research, including patient-directed research goals and the inclusion of patient-reported outcome measures [3, 4]. In line with this goal, early collaborative efforts of the SLSMDS Research Network resulted in the development of a patient registry and natural history study [5]. These patient-led research infrastructures intended to improve clinical care, address gaps in clinical trial readiness, and identify logical endpoints that are meaningful for patient quality of life.

Building upon these research infrastructures, The Champ Foundation and CHOP jointly hosted the SLSMDS Family and Scientific Conference in July 2022. The conference was attended by clinicians, researchers, and families and afforded an opportunity to collect cross-sectional data in a rare disease community whose affected individuals are difficult to access, characterize, and report on. We describe our data collection procedures, as well as the laboratory and patient-reported outcome results from the conference research study. Ultimately, we demonstrate how effective patient-research partnerships and conference data collection can serve as a reproducible model for the rare disease community to overcome common obstacles of small patient numbers, geographic spread, and unclear clinical endpoints.

### Patient-research partnerships and SLSMDS research infrastructures

#### CFR registry

The Champ Foundation Registry (CFR) is a registry of individuals with SLSMDS established in 2020. The goals of the CFR are to enhance the understanding of SLSMDS through patient reported outcomes (PROs) and improve understanding of the natural history of the disease [6, 7]. The CFR was developed in collaboration between The Champ Foundation, researchers from SLSMDS Centers of Excellence including CHOP, and feedback from families of affected children [5]. Table 1 outlines specific roles of The Champ Foundation and CHOP to develop and maintain the registry. Additional information on the registry’s development and design, as well as preliminary findings, have been described [5, 8].

**Table 1:** Patient-researcher partnerships in the development and maintenance of SLSMDS research infrastructures

Research infrastructure	Role of the champ foundation (patient advocates)	Role of CHOP (researchers and clinicians)
Champ Foundation Registry (CFR) *Launched August 2020; recruitment ongoing*	• Financial supporter • Hosted 2020 conference to launch SLSMDS Research Network and establish registry goals • Engage all stakeholders to get feedback on registry development, including researchers, clinicians, and families • Responsible for centralized IRB • Lead recruitment efforts via website, social media	• Participated in the registry working group • Reviewed and finalized all survey modules • Shared CFR with existing patients • Data analyses and publications
SLSMDS Natural History Study *Launched for PS patients in September 2021; expanded to all SLSMDS patients in October 2022; Data collection ongoing*	• Financial supporter • Identified patient-priorities in online community calls • Centralized IRB • Lead recruitment efforts via CFR, website, and social media • Coordinate and pay for family travel (flights and hotels) • Data management on the CFR	• Determine standardized assessments and endpoints • Coordinate patient appointments and provide clinical care to participants • Local IRB and consenting • Report outcomes back into the registry • Data analyses and publications
Conference data collection *Single day data collection in July 2022*	• Financial supporter • Centralized IRB • Lead recruitment efforts via CFR, website, and social media • Coordinate and pay for family travel (flights and hotels) • Responsible for data management on the CFR	• Host conference at location • Determine standardized assessments and endpoints • Local IRB and consenting • Coordinate phlebotomist and research coordinator • Report outcomes back into the registry • Data analyses and publications

#### SLSMDS natural history study

The SLSMDS Natural History Study aims to collect data on standardized clinical outcomes and help answer patient- and clinician-identified questions regarding the disease, including its causes and potential treatments. The SLSMDS Natural History Study takes place at two SLSMDS Centers of Excellence: the Cleveland Clinic and CHOP. The study began recruiting in 2021 and data collection is ongoing with financial support for travel for visits from The Champ Foundation (Table 1).

### Role of rare disease conferences

Rare disease conferences provide opportunities to define the current state of disease-specific research, disseminate preliminary findings, and identify future research collaborations [9]. Patient and family attendance at conferences is beneficial for sharing priorities in the development of standards of care [10], contributing to discussions on clinical trial endpoint determination [11], and vocalizing care concerns to researchers and clinicians [12]. This is particularly important given the Food & Drug Administration (FDA) increased emphasis on clinically meaningful endpoints for clinical trials [13]. Patient and family input is essential to determine the meaningfulness of those endpoints.

Conferences also present a unique opportunity for families of affected children to gather. Often in rare disease communities, families are geographically dispersed and infrequently meet someone with the same disorder. In-person meetings can provide emotional support to the families [11, 14–16]. Being able to simultaneously see many affected individuals is also a luxury for the rare disease clinicians and provides a unique opportunity to identify disease features that may not be as striking when seen in individual patients [17]. Despite these advantages, few previous publications have outlined either patient-reported outcomes or phenotype advancements from rare disease conferences.

Finally, joint family-clinician conferences present an ideal opportunity for data collection and research, especially in the rare disease space. To date, only limited formalized research studies have been conducted and reported on in conference settings. Silier et al. [18] described data collected at a family conference from pediatric patients with chronic non-bacterial osteitis. In another, Strom et al. [19] described a carrier screen conducted at a meeting for individuals and their families affected by Tay-Sachs disease, where a first or second degree relative could volunteer to provide a blood sample for genetic sequencing. The Batten Disease Support and Research Association (BDSRA) designated their annual conference as a “mobile clinical research laboratory” designed to facilitate sample data collection and reduce family travel burdens [20]. Data collected at their conference included neuropsychological assessments and buccal epithelial cell collection. The Cystinosis Foundation conducted a focus group research study during their family conferences to better understand the experience of living with cytinosis in emerging adulthood [15]. These studies demonstrate feasibility and utility of rare disease data collection in a conference setting.

### Current study

Here we describe collaborative efforts to host the SLSMDS Family and Scientific Conference and outline collection procedures for laboratory data and patient reported outcomes and share generated data. We aim to demonstrate how patient-researcher partnerships can develop and sustain mechanisms to collect data on rare and ultra-rare diseases, where patients are generally geographically scattered and opportunities for standardized data collection are limited. Ultimately, this type of conference is a valuable pathway for improving our understanding of the natural history of rare disorders and ultimately supporting development of future treatments.

## Methods

### Procedure

The SLSMDS Family and Scientific Conference was primarily advertised via email and social media by The Champ Foundation and CHOP. A few months before the conference, CFR participants were directly notified by email and invited. Day 1 was an informal picnic at the Philadelphia Zoo for families, researchers, and clinicians to facilitate relationship building. Day 2 was a formal scientific event, with presentations by leading researchers, clinicians, and scientists. In addition, during Day 2, select families were invited to give short presentations about their experience with SLSMDS. Families were invited to participate in one or both days, and a virtual option was available to participate on Day 2 via Zoom. Accommodations and travel for families was supported by The Champ Foundation. There were no registration fees.

When registering, caregivers or affected individuals could consent to be contacted for participation in research during Day 2 of the conference. If they agreed, a research coordinator from CHOP contacted them to collect informed consent to participate in the research study and emailed them advance copies of electronic surveys to reduce survey fatigue at the conference.

At the conference, the designated area for research included an intake assessment table and a private phlebotomy station. If not completed ahead of time, the family could complete the research consent and questionnaires in person. Families were also offered SLSMDS Natural History Study (NHS) and/or clinical care appointments the day before the conference. Individuals not yet enrolled in the SLSMDS NHS were offered enrollment. For clinical participants, blood samples for conference research, SLSMDS NHS and/or clinical care were collected simultaneously to avoid multiple needle sticks. All research blood draws were completed within 1–3 days of the conference date. Results of research lab tests were only returned to the patient if they were determined by the study principal investigator to be clinically actionable. Table 1 describes the patient-research partnership critical for facilitating conference data collection.

Following the conference, all conference attendees were invited by email to complete an online survey and provide feedback on the conference. For example, one question asked “What was your favorite part of the conference?” Responses to this question are presented in an additional file [See Additional File 1].

### Participants

**Fig. 1:**
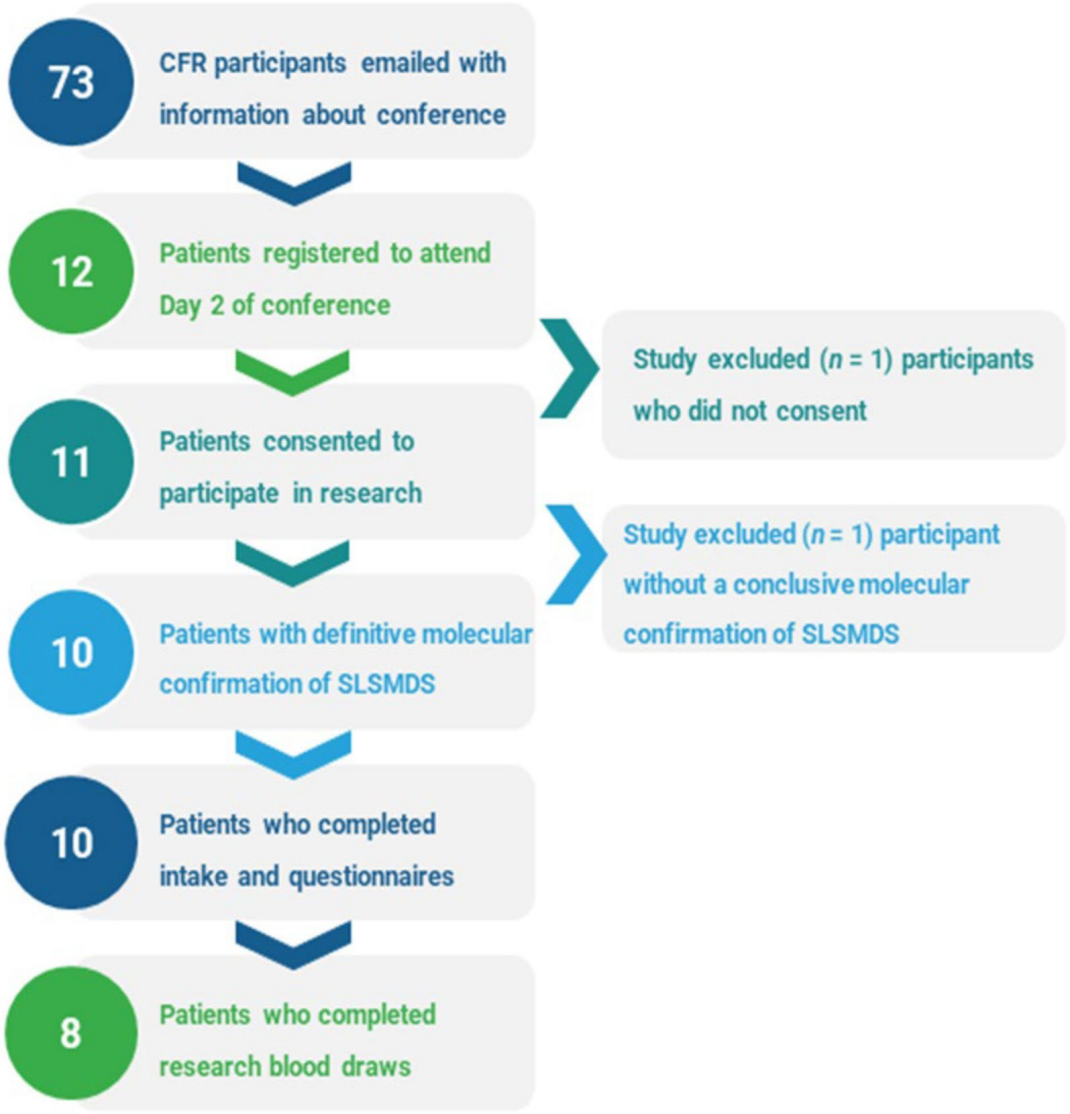
Participant inclusion and completed assessments. Schematic outlining the process of conference registration and research consent. The number of participants involved at each stage is indicated to the left of the description

The inclusion and exclusion criteria for the participants are presented in Fig. 1. 73 families of affected individuals from the CFR were invited by email to attend the conference and participate in research. 12 families of affected individuals registered for the in-person portion of the conference. Of those 12 individuals, 11 consented to the research study. The data were analyzed from individuals with definitive molecular confirmation of a SLSMDS (n = 10). All conference research participants were also CFR participants.

### Measures

Survey questionnaires and laboratory assessments were collected for the present study.

#### Questionnaire measures

##### Demographic and intake questionnaire

The intake form asked families to self-report on the patient’s diagnosis (Pearson Syndrome, KSS, CPEO or SLSMDS not otherwise specified (SLSMDS-NOS)), transfusion dependency (yes/no), weight, height, last enteral intake (time), parenteral nutrition status (yes/no), current interventions (e.g.., whether the patient was currently taking growth hormone (yes/no), and duration of growth hormone use, if applicable). Z-Scores for height calculations were derived from the population means reported by the U.S. Department of Health and Human Services Centers for Disease Control (CDC) [21]. The complete demographic and intake questionnaire is provided in an additional file [see Additional File 2].

##### ***EQ-5D-Y ***[22]*** (youth, ages 0–15 years) parent and child-report versions***

This scale is used to measure health-related quality of life across five dimensions: (1) mobility, (2) looking after oneself, (3) doing usual activities, (4) having pain or discomfort, and (5) feeling worried, sad, or unhappy. The EQ-5D-Y also includes a Visual Analog Scale (EQ VAS) for a rating of overall current health on a scale of 0–100, where 0 is ‘The worst health you can imagine’ and 100 is ‘The best health you can imagine’.

##### ***Lansky scale ***[23]*** (ages 0–16 years)***

This scale is designed to assess general functional status and play-performance for children aged 15 years and younger. Respondents are given options on a scale from 10 to 100 and are asked to respond in increments of 10 as to their or their child’s activity status over the past month, where 100 at the top of the scale indicates “Fully active” and 10 at the bottom of the scale indicates “Completely disabled, not even passive play”. The complete scale is provided in an additional file [see Additional File 3].

##### ***Quality of life in neurological disorders (neuro-QOL) pediatric anxiety scale ***[24]*** (ages 8–17 years)***

The Neuro-QOL battery was designed to quantify specific dimensions of health-related quality of life in children with neurological disorders, including muscular dystrophy and epilepsy. The Neuro-QOL Anxiety bank specifically evaluates pediatric anxiety. Scores are presented as T-Scores with a general reference mean of 50 and a standard deviation of 10.

##### ***The multidimensional anxiety scale for children second edition (MASC-2) ***(25)***(ages 8–19 years) parent and self-report versions***

The MASC-2 is designed to assess several domains related to childhood anxiety including separation anxiety and phobias, social anxiety, obsessions and compulsions, physical symptoms, harm avoidance, and the Generalized Anxiety Disorder (GAD) Index. Scores were normed on a T-scale (Mean (M) = 50, Standard Deviation (SD) = 10) based on normative data in three age categories: 8–11 years, 12–15 years, and 16–19 years (none of the latter category in our study cohort). Mental health resources were offered to the family upon completion of this scale.

#### Laboratory measures

The laboratory measures included a complete blood count (CBC), a comprehensive metabolic panel (CMP), cystatin C, fructosamine, lactate, growth differentiation factor 15 (GDF-15), reticulocyte count, and ionized calcium.

### Statistical analyses

Descriptive statistics were derived using Tableau Version 2022.4.0. A Pearson correlation was used to evaluate the relationship between the two measures of overall health status (Lansky Score and EQ-5D-Y VAS) and the correlation coefficient was calculated using GraphPad Prism version 9.3.1 with significance reported at the < 0.01 level.

## Results

### Demographics and intake

All participants (n = 10) completed the demographics and intake questionnaire (Fig. 1). Participants ranged in age from 2 to 15 years (987 to 5,693 days; M = 3,749 days, SD = 1,489 days) and n = 3 patients were female (30%). At the time of intake n = 5 participants (50%) reported a diagnosis of PS, n = 4 participants reported a diagnosis of KSS (40%), and n = 1 (10%) participant reported a diagnosis of SLSMDS NOS. Two participants reported that they were currently taking growth hormone but only one reported duration of treatment (five years). One participant was transfusion dependent at the time of intake and no participants reported any parenteral nutrition at the time of intake.

**Fig. 2:**
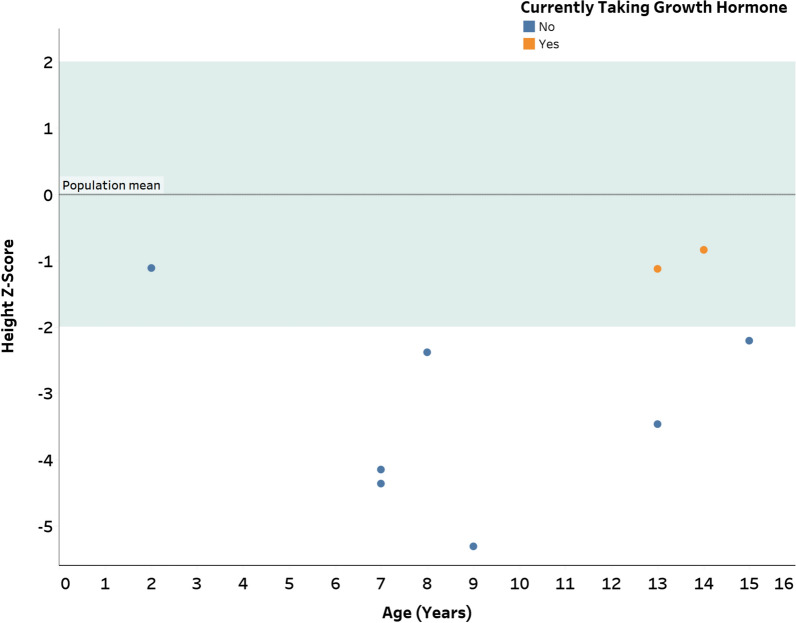
Z-scores for height. Z-scores were calculated using sex, age, height, and population averages from the U.S. Department of Health and Human Services Centers for Disease Control and Prevention. A Z-Score of zero represents the population mean and the shaded region indicates values that fall within 2 standard deviations above or below the population mean

Z-scores for self-reported height for n = 9 participants at the time of intake were calculated and ranged from -5.31 to -0.83 (M = − 2.78, SD = 1.62, Fig. 2). The Z-score for height was less than 2 standard deviations of the normative mean of zero for 6 participants (67%). The two patients taking growth hormone ranked among the three highest z-scores for height in the cohort (− 0.83 and − 1.13, along with one other participant with a height z-score of -1.11, who was not reportedly taking growth hormone).

### Questionnaire results

All participants (n = 10) completed questionnaires relevant to their age category (Fig. 1). The EQ-5d-Y self-report versions (n = 2) and EQ-5d-Y Parent-Report versions (n = 8) revealed that most problems were reported in the Mobility and Self-Care (also titled “Looking After Oneself”) domains (Table 2).

**Table 2:** EQ-5D-Y dimension and severity

EQ-5D-Y dimension		Responses n (0%)
Mobility	No problems	2 (20%)
	Some problems	8 (80%)
	A lot of problems	0 (0%)
Looking after oneself	No problems	2 (20%)
	Some problems	8 (80%)
	A lot of problems	0 (%)
Doing usual activities	No problems	4 (40%)
	Some problems	6 (60%)
	A lot of problems	0 (0%)
Having pain or discomfort	No problems	7 (70%)
	Some problems	3 (30%)
	A lot of problems	0 (0%)
Feeling worried, sad or unhappy	Not worried, sad or unhappy	4 (40%)
	A bit worried, sad or unhappy	6 (60%)
	Very worried, sad or unhappy	0 (0%)

EQ-5D-Y data, showing frequencies and proportions by dimension and severity level. Responses are expressed as the number of participants providing that response as well as the percentage of the total number of participants selecting that response.

**Fig. 3:**
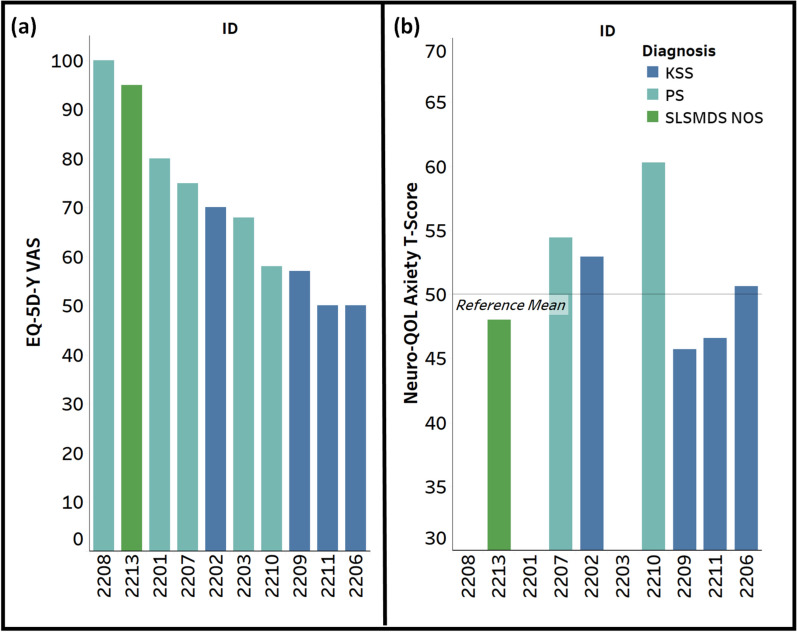
EQ-5D-Y visual analog scale (EQ VAS) rating and neuro-QOL anxiety T-score. **a** EQ-5D-Y VAS Rating ranges from 0 (“worst health you can imagine) to 100 (“best health you can imagine”); **b** Neuro-QOL Anxiety scale T-Score with a normative mean of 50 and standard deviation of 10

Scores on the EQ VAS (n = 10) ranged from 50 to 100 (M = 70.3, SD = 17.5), where 100 is “The best health you can imagine” and 0 is “The worst health you can imagine” (Fig. 3a).

The Neuro-QOL Pediatric Anxiety Scale is intended for children aged 8 years or older and was completed by n = 7 participants who were 8 years or older at the time of completion. T-Scores on the Neuro-QOL ranged from 45.7 to 60.3 (M = 51.2, SD = 5.13; Fig. 3b), where a score of 50 represents the average of the general reference population and a score of 60 represents one standard deviation above the general reference population (e.g. worse anxiety).

Scores for the Lansky scale ranged from 50 (n = 2) to 100 (n = 1). 40% of this cohort (n = 4) rated a Lansky score of 70 and the remaining 2 participants rated a Lansky score of 80. Lansky Score and EQ-5D-Y VAS, which are both indicators of overall health status, were strongly and significantly correlated (r = 0.78, *p* < 0.01).

**Fig. 4:**
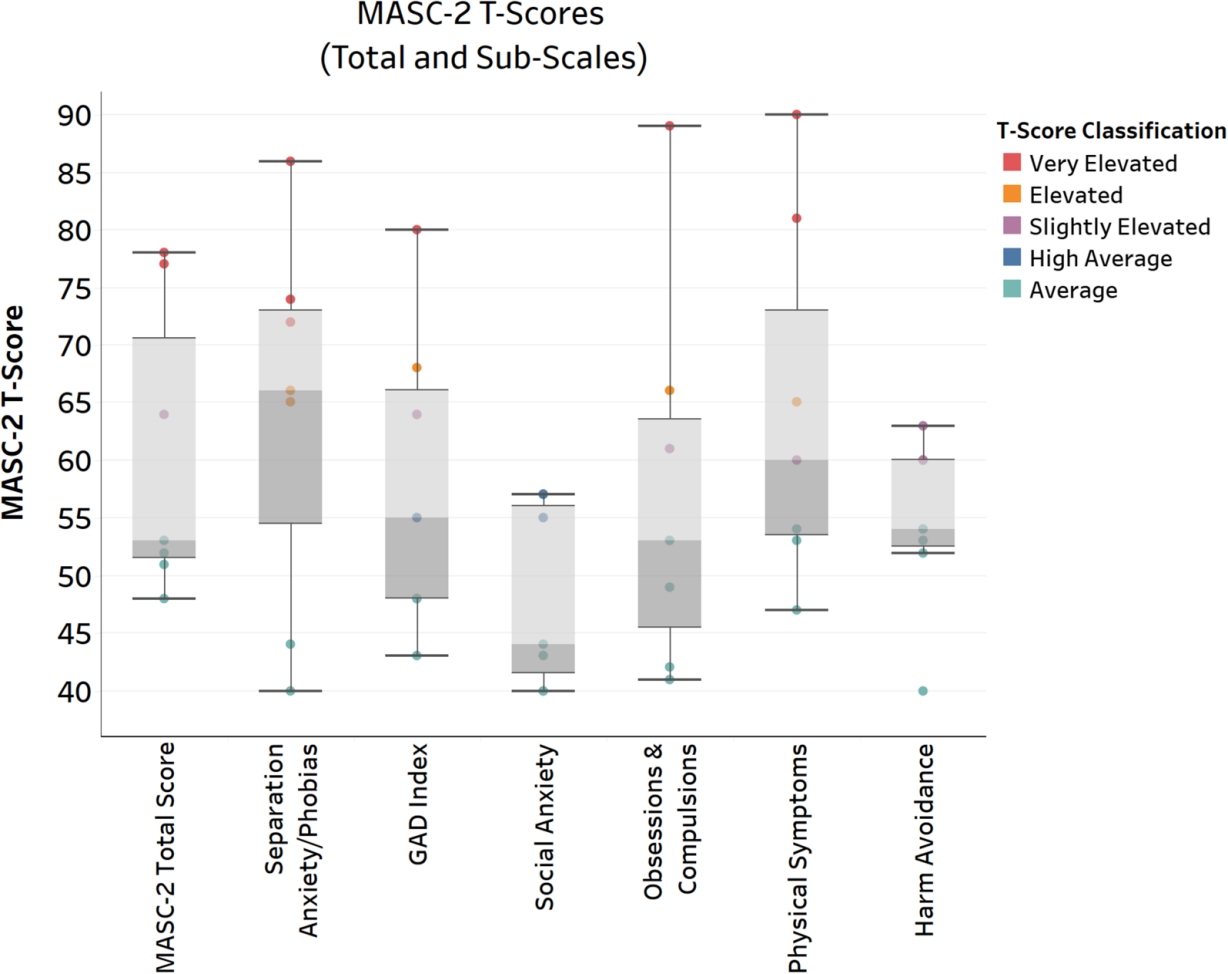
MASC-2 total and sub-scale T-scores. Scores are normed on a T-Scale with a mean of 50 and standard deviation of 10. Scores are colored by T-Score severity classification

The MASC 2 questionnaire is intended for children 8 years or older, and was completed by n = 7 participants who were 8 years or older at the time of completion. One of the 7 participants completed the MASC 2 questionnaire through self-report. MASC 2 scores showed wide variability in our cohort, ranging from “Average” (within 1.5 standard deviations of the normative mean) to “Very Elevated” (e.g. 2–4 standard deviations above the normative mean; Fig. 4). In this cohort the highest average anxiety ratings were in the domains of Separation Anxiety/Phobias (*M* = 64.4, *median* = 66) and Physical Symptoms (*M* = 67.5, *median* = 60). The physical symptoms domain is made up of two subscores: Panic (*M* = 69.4, *median* = 67) and Tense/Restless (*M* = 63.75, *median* = 56). Of the 7 participants with MASC 2 scores, n = 2 were classified as having a “Low Probability” anxiety classification, n = 2 as having “Borderline Probability” anxiety classification, and n = 3 as having a “High Probability” anxiety classification.

### Laboratory results

Research blood draws were completed by 8 participants (Fig. 1) although lactate tests were cancelled for two participants due to a procedural error. All 8 patients had elevated GDF-15 levels above the reference value of 750 pg/mL. Five of the six participants had lactate values above the upper limit of normal (2 mmol/L). Two patients had glucose levels out of the reference range: one patient was hyperglycemic and one patient was hypoglycemic. One patient had abnormal glycemic control, as demonstrated by elevated fructosamine, which was not previously known. One patient with known hypoparathyroidism had clinically significant hypocalcemia. Finally, three patients with known chronic kidney disease had elevated cystatin C, indicating impaired glomerular function. All laboratory results are provided in Table 3.

**Table 3:** Laboratory results

ID	Age (Years)	Diagnosis	GDF-15 (pg/mL)	Lactate (mmol/L)	Glucose (mg/dL)	Fructosamine (umol/L)	Ionized calcium (mg/dL)	Cystatin C (mg/L)
2208	2	PS	**5478**	**9.4**	101	211	10.1	0.53
2201	7	PS	**5178**	–	86	279	**8**	**1.31**
2203	7	PS	** > 6000**	**3.8**	102	**326**	10.6	**2.2**
2210	8	PS	** > 6000**	**2.2**	**65**	239	9.5	**4.95**
2202	9	KSS	**4917**	**3.2**	92	257	10.7	0.83
2206	13	KSS	**1799**	**3.1**	103	281	9.4	0.71
2213	14	SLSMDS-NOS	**1578**	1.3	86	272	9.7	0.71
2209	15	KSS	**1584**	–	**142**	**295**	9.4	0.74

Research blood test results

Bolded values indicate abnormal value relative to the reference range

## Discussion

The SLSMDS Family and Scientific Conference was a result of an existing strong collaboration infrastructure and enabled cross-sectional data collection on multiple SLSMDS patients simultaneously. Using patient- and family-reported data, we expand our understanding of the psychiatric manifestations of SLSMDS, self-care and quality of life, and present the template of the SLSMDS Family and Scientific Conference as a general model of value to the rare disease community.

These results generated at the conference corroborate previous CFR findings that psychiatric disturbances are recurrent in patients with SLSMDS [5]. The majority of the participating children reported feeling sad, worried, unhappy, and experiencing physical symptoms of panic and separation anxiety. Psychiatric symptoms had not been appreciated as a symptom of SLSMDS prior to the incorporation of family-led research priorities and patient-reported outcome measures. Research on other pediatric disorders has also revealed correlations between multi-systemic and progressive disorders and psychiatric problems [26]. Psychiatric symptoms are likely under-ascertained in multi-systemic genetic diseases in general, and SLSMDS in particular. These findings suggest that it is critical to systematically assess for mental health symptoms. Multi-disciplinary teams should incorporate mental health specialists to support children and families, and offer best practice treatments and solutions (e.g., minimize separation, calming strategies).

There was variability in reported levels of self-care and mobility. Some affected individuals and caregivers reported no-to-few problems with mobility, looking after themselves, and/or doing usual activities. Additionally, many respondents said the affected individual had no problems with pain or discomfort. Indeed, no participant responded “a lot of problems” across any of the EQ-5D-Y scales. Given the multi-systemic and progressive nature of SLSMDS, this is surprising. Previous studies [27, 28] have emphasized the significant functional impact of mitochondrial disease. Given the spectrum of SLSMDS, there was likely some self-selection of conference attendees. Children who were well enough to travel and participate in a multi-day conference may be significantly different from children who were not well enough to travel or participate. However, the overall more positive than anticipated quality of life responses emphasize the importance of utilizing systematic measures of quality of life in research assessments.

Our findings on anthropomorphic data are consistent with previous research reporting failure to thrive, short stature, and low BMI for children with [29, 30] SLSMDS. As expected, participating children were smaller than their peers, according to the normative data shared by the CDC. The role of growth hormone for SLSMDS patients has not been extensively reported in the literature. We found that the patients on supplemental growth hormone ranked among the three highest z-scores for height, and had near-normal heights for age. This observation comes from a small number of patients, but suggests potential benefit of more wide-scale growth hormone use.

The laboratory findings emphasize the potential use of GDF-15 as an important biomarker for children with mitochondrial disease and SLSMDS. GDF-15 is a known mitochondrial disease biomarker that is particularly elevated in mitochondrial myopathies [31, 32]. However, it has limited sensitivity to mitochondrial diseases as a whole [33, 34], therefore the fact that it was universally elevated in all participants, suggesting that it is highly sensitive for SLSMDS, was striking. Lactate has less sensitivity compared to GDF-15.

Three out of eight children had elevated Cystatin C, a marker of kidney function. While some research has shown that SLSMDS can cause glomerular dysfunction, it may be underappreciated as a sign, since creatinine, a more conventional marker of glomerular function, is less sensitive in patients with low muscle mass [35], as seen in many patients with SLSMDS. Glomerular dysfunction is a major cause of morbidity and mortality. Therefore, screening with cystatin C is an important factor to consider for future clinical care. Future research on kidney function and SLSMDS is warranted to support clinical recommendations for monitoring and treatment. Similarly, we identified one patient with elevated fructosamine who was not previously known to have impaired glycemic control. The more common screening test for impaired glycemic control is hemoglobin A1c, which has lower sensitivity in PS due to rapid hemoglobin turnover and red blood cell transfusion dependence [36]. These two examples emphasize the importance of systematic screening performed by centers with disease-specific expertise.

Overall, we demonstrate that patient data collection at rare disease conferences is feasible and provides valuable data particularly on patient-reported outcomes and patient and family priorities for future therapies. An unexpected benefit of the conference research setting was the opportunity for clinicians to informally observe multiple children with the same rare disease and develop emergent observations. It is unusual for clinicians to be able to see many patients with the same rare disease in a single day, and it is unusual for patients with the same rare disease to interact with each other. This allowed clinicians, patients, and families to notice patterns that are not immediately recognizable when seeing patients individually. For example, clinicians noticed an almost universal presentation of decreased facial expression among SLSMDS patients of all ages. We are now evaluating methods for objectively and subjectively assessing facial expressions for additional study into our ongoing longitudinal natural history study assessments. Such hypothesis-generating and emergent observations are a unique benefit to the setting. Future directions include improving conference infrastructure to facilitate such observations. Furthermore, conference participants reflected on the benefits of the collaborative experience, highlighting themes such as interpersonal connection and social support, education, cooperation, and shared goals.

## Limitations

Our results demonstrate the possibility and potential of data collection at rare disease conferences. However, specific limitations are important to note. This population is not fully representative of children with SLSMDS. The participants in this research study were well enough to travel for a 2 day conference. For example, the organizers are aware that some non-participating children were unable to travel from their home hospital due to difficulties with breakthrough seizure management and/or overall fragility. We can speculate that patients with infection precautions, frequent intravenous treatments (such as transfusions) or cardiopulmonary instability may also not have been able to attend. Future conferences using this as a model for data collection should utilize strategies to examine differences between the group who is able to travel and participate and those who are not able. Additionally, even though family hotels and flights were supported by The Champ Foundation, conference-based ascertainment biases towards patients of higher socioeconomic status because of the required time off for travel, and/or caregiving responsibilities for other relatives. Previous research has found the CFR sample to be disproportionately affluent and well-educated compared to the general population, suggesting that children from less affluent families are underrepresented in the registry and were less likely to be recruited to participate in the conference research study. Further, we acknowledge that funding this type of conference is challenging for small patient-led nonprofit organizations. The Champ Foundation’s ability to initiate this conference was made possible through a Chan Zuckerberg Initiative grant, which is a unique funding model for patient-led organizations. Additional limitations include the small sample size and that this was a single timepoint cross-sectional study. Ongoing and future collaborations between researchers, clinicians, and advocacy groups should prioritize longitudinal study design.

## Conclusions

Patient-research partnerships between The Champ Foundation, a parent-run patient advocacy group, and CHOP, a SLSMDS Center of Excellence, enabled novel data collection from individuals with ultra-rare mitochondrial disease. This paper describes single-time point laboratory assessments and patient reported outcomes for a subset of individuals with SLSMDS. The results relating to multi-systemic disease and the potential use of biomarkers confirm the findings of previous studies. Emergent findings such as psychiatric burden highlight the unmet medical and social needs of individuals with SLSMDS. In summary, efficient patient-researcher partnerships can develop and sustain novel mechanisms to collect rare disease data, improve our understanding of the natural history of the disorders, and ultimately support development of future treatments.

## Supplementary Information

Additional file1 Conference Feedback. List of select responses to the question posed to conference attendees, including parents of affected children, clinicians, and researchers: “What was your favorite part of the conference?”.

Additional file2 Demographic and Intake Questionnaire. Full demographic and intake questionnaire as completed by research participants.

Additional file3 Lansky scale. Full Lansky scale as completed by research participants.

## Data Availability

The datasets used and/or analyzed during the current study are available from the corresponding author on reasonable request.
