# Supplementary material for: Single large-scale mitochondrial DNA deletion syndromes: scientific and family conference optimizes the collection of rare disease research outcomes
Source: Orphanet J Rare Dis. 2025 Aug 4;20:399. doi: 10.1186/s13023-025-03632-4 (PMC12323275; doi:10.1186/s13023-025-03632-4)
Supplement: Supplementary file 1 — Additional file1 Conference Feedback. List of select responses to the question posed to conference attendees, including parents of affected children, clinicians, and researchers: “What was your favorite part of the conference?”. [file 13023_2025_3632_MOESM1_ESM.docx]

`

All SLSMDS Science and Family Conference attendees were emailed a follow up survey the week after the conference was held. Here are select responses to the question, **“What was your favorite part of the conference?”**

| **Parents of affected children** | - Honestly, just being around other families that truly “get it.” Having a child with a rare disease can be extremely isolating. We didn't get to speak to everyone but there was a connection that we felt with all the families and even doctors that was on a entirely different level - Meeting other families affected by PS and/or SLSMDS - Being able to meet and interact with other families going through the same struggles! - Seeing all the families together...the group photo...that we are not alone and can make an impact together - The fact that it was casual enough that kids could be coming in and out yet professional enough that the researchers shared their projects in detail - Learning about the clinical trials and research underway - So many doctors and families in the same room with the same goal! |
| --- | --- |
| **Clinicians** | - Meeting with all the families especially socially - The day in the Zoo. Meeting parents and their children. Very inspiring and informative. - The patient’s families lectures |
| **Researchers** | - The children and their families - Meeting / reconnecting with children and families - Engaging with the patient families and the research talks - Getting to speak with patients and families! - Joining the family’s together with world-class scientists - Hard to pick a favorite part. The patient stories were moving, the science was inspiring, the community was uplifting. - I loved hearing the patient stories. I am a new hire…and I am not super familiar with SLSMDS, so getting to hear about different perspectives and meeting the families was very inspiring to me. - I enjoyed both the research progress/plan and patient stories – the latter are truly motivating |
