# Supplementary material for: Single large-scale mitochondrial DNA deletion syndromes: scientific and family conference optimizes the collection of rare disease research outcomes
Source: Orphanet J Rare Dis. 2025 Aug 4;20:399. doi: 10.1186/s13023-025-03632-4 (PMC12323275; doi:10.1186/s13023-025-03632-4)
Supplement: Supplementary file 2 — Additional file2 Demographic and Intake Questionnaire. Full demographic and intake questionnaire as completed by research participants. [file 13023_2025_3632_MOESM2_ESM.docx]

**Additional File 1: Demographic and Intake Questionnaire**

Record ID _________

Date of blood draw __________

Time of blood draw (HH:MM)

Age in days _________

Diagnosis _________

- Pearson syndrome
- Kearns-Sayre syndrome (KSS)
- CPEO
- SLSMDS NOS

Is the patient transfusion dependent?

- Yes
- No
- I don’t know

Weight (self-reported; lbs) _________

Weight (self-reported; kg) _________

Height (self-reported; feet) _________

Height (self-reported; inches) _________

Height (self-reported; cm) _________

Last enteral intake (time they ate last, either by mouth or tube feeding) _________

Any parenteral nutrition? (intravenous administration of nutrition)

- Yes
- No
- I don’t know

Is the patient currently taking growth hormone?

- Yes
- No

Duration of treatment with growth hormone _________

Contact method for clinical results to be returned

- Email
- Phone

Email address for results _________

Phone number for results _________
