# Supplementary material for: Single large-scale mitochondrial DNA deletion syndromes: scientific and family conference optimizes the collection of rare disease research outcomes
Source: Orphanet J Rare Dis. 2025 Aug 4;20:399. doi: 10.1186/s13023-025-03632-4 (PMC12323275; doi:10.1186/s13023-025-03632-4)
Supplement: Supplementary file 3 — Additional file3 Lansky scale. Full Lansky scale as completed by research participants. [file 13023_2025_3632_MOESM3_ESM.docx]

**Additional File 2: Lansky scale**

*Please review the scale below and select the score (10-100) that best represents your child’s activity status over the past month.*

**Lansky Scale (age 0-16)**

| Able to carry on normal activity; no special care needed | |
| --- | --- |
| 100 | Fully active |
| 90 | Minor restriction in physically strenuous play |
| 80 | Restricted in strenuous play, tires more easily, otherwise active |
| Mild to moderate restriction | |
| 70 | Both greater restrictions of, and less time spent in, active play |
| 60 | Ambulatory up to 50% of the time, limited active play with supervision/ assistance |
| 50 | Considerable assistance required for any active play, fully able to engage in quiet play |
| Moderate to severe restriction | |
| 40 | Able to initiate quiet activities |
| 30 | Needs considerable assistance for quiet activity |
| 20 | Limited to very passive activity initiated by others (e.g. TV) |
| 10 | Completely disabled, not even passive play |
